# Environmental Lead after Hurricane Katrina

**DOI:** 10.1289/ehp.1104909

**Published:** 2012-05-01

**Authors:** Howard W. Mielke

**Affiliations:** Tulane University School of Medicine, New Orleans, Louisiana, E-mail: hmielke@tulane.edu

I support the warning from Rabito et al. (2012) about the hazards of power sanding and their call for enforcing the New Orleans, Louisiana, Code of Ordinances (2001) and U.S. Environmental Protection Agency (EPA) Renovation, Repair, and Painting Rule (U.S. EPA 2008) for cleanup of residual lead dust at residential properties. Power sanding has been a scourge to the safety and well-being of children and pets in New Orleans for many years (Jacobs et al. 2003; Mielke et al. 2001).

Unfortunately, Rabito et al. (2012) made a fundamental error by comparing medians from soil data (Mielke et al. 2005), which I shared with them, with principally foundation soil data. These medians included four sample locations: foundation, open space (i.e., mid-yard), residential-street side, and busy-street side. Comparing pre-Katrina median soil lead from our survey with foundation soil samples post-Katrina does not provide insight into foundation soil lead changes stemming from renovation.

Furthermore, evidence from Louisiana blood lead surveillance shows decreasing—not increasing—exposure after Hurricane Katrina. The 2008 Orleans Parish results show that 38.5% of the children were tested, and 6.4% of these presented with lead concentrations ≥ 10 µg/dL (Louisiana Childhood Blood Lead Surveillance System 2009). In 2010, 40.0% of the children in Orleans Parish were tested, including a higher proportion of children from outer areas of New Orleans, and 3.3% presented with ≥ 10 µg/dL (Louisiana Childhood Blood Lead Surveillance System 2010). The continuing pattern of lead poisoning is uneven, with larger preva-lence in the interior of the city and lower prevalence in outer areas of the city (Zahran et al. 2011, their Figure 1). From the perspective of lead changes in soil and blood, both measurements decreased after Katrina (Zahran et al. 2010). Although large numbers of children are still at risk from lead poisoning, the blood lead trends reported for New Orleans do not support the future implications suggested by Rabito et al. (2012).

Regarding the future, it is important to note that New Orleans is under-taking improvements to playgrounds so children will have lead-safe play areas in every neighborhood (Schleifstein 2011). A project is also under way to improve the quality of play areas at New Orleans child care centers (Mielke et al. 2011a). In addition, projects to eliminate lead-based paint and soil lead hazards at public housing properties are ongoing (Reckdahl 2011). The implications are that in the future, New Orleans will be safer with respect to lead. Private residential properties often have higher risk for lead poisoning than do public properties (Mielke et al. 2011b), and Rabito et al. have appropriately called attention to the need to address lead contamination of private residential properties.
